# The effect of vestibular, lingual, and aligner appliances on the quality of life of adult patients during the initial stages of orthodontic treatment

**DOI:** 10.1186/s40510-020-00346-0

**Published:** 2021-01-18

**Authors:** Maryam AlSeraidi, Ismaeel Hansa, Fadia Dhaval, Donald J. Ferguson, Nikhilesh R. Vaid

**Affiliations:** 1 Department of Orthodontics, European University College, Dubai, United Arab Emirates; 2 Durban, South Africa; 3 Mumbai, India

**Keywords:** Quality of life, Adult, Lingual appliance, Vestibular appliance, Clear aligners

## Abstract

**Background:**

Patient quality of life (QoL) during orthodontic treatment is an important consideration that requires greater academic investigation as greater focus is placed on enhancing patient experience. Quality of life (QoL) was assessed in three orthodontic appliance groups, i.e., vestibular, lingual, and aligners during the initial stages of treatment. The sample was comprised of 117 adult patient-subjects distributed into 3 groups: vestibular (*n* = 41), lingual (*n* = 37), and aligner (*n* = 39). A WHOQOL-BREF questionnaire surveyed four domains (physical health, psychological health, social relationships, and environment).

**Results:**

Mean scores for domain 1, physical health, showed that the aligner group (28.1) had significantly greater scores than that of the vestibular (22.7) or lingual (22) groups. Domain 2, psychological health, demonstrated significant differences (*P* < 0.001) between all groups, with the aligner group scoring the highest (23.2), followed by the lingual (18.4) and vestibular (15.2) groups. Domain 3, social relationship, showed that aligner (10.9) and lingual (10.2) scores were significantly greater (*P* < 0.001) than those of the vestibular group (7.8). Domain 4, environment, displayed significant differences between all groups, with the aligner group scoring highest (32.1), followed by the lingual group (29.3), and lastly the vestibular group (26.4). Overall, the highest mean score was obtained by the aligner group (23.1) and the lowest mean score was by the vestibular group (18). The mean domain scores for all three groups were significantly different (*P* ≤ 0.005) from each other (Table 2).

**Conclusions:**

Overall, patients undergoing Aligner therapy reported the overall highest QoL scores, followed by lingual and vestibular groups.

## Introduction

The traditional goals of orthodontics are esthetics, functional stability, and structural harmony. Patient quality of life (QoL) and their orthodontic journey, however, is also an important consideration that is now under greater academic investigation. From the end of the 19th century, fixed appliances were most commonly used in America, while removable appliances were favored in Europe. This continued until the introduction of stainless steel and bonding adhesives. “Metal mouth” or “train tracks” are common terms used to describe the appearance of patients wearing traditional metal appliances. Even today, traditional vestibular appliances are still the main stay of orthodontics. The introduction of lingual appliances in the late 1970s provided a significant esthetic alternative to patients [1, 2]. The main disadvantages were higher laboratory costs and technical limitations of both the appliances and operators. Concealed behind the teeth, they are most often worn by adult patients who are conscious about their image [3]. Another great benefit of lingual appliances is that they are less likely to cause white spot lesions than conventional vestibular appliances [4]. Studies have found no differences in adaptation time between vestibular and lingual appliances, with both appliances needing about a month to adjust. Lingual patients did report greater speech disturbances and irritation of the tongue, while pain was similar between the two appliances [5–7]. Both appliances result in similar levels of treatment satisfaction according to Wu et al. [8].

Invisalign™ revolutionized the esthetic treatment terrain by popularizing clear aligner treatment [9]. The esthetic advantage of clear aligners has been well documented and may well ameliorate the social anxiety related to orthodontics. Invisalign™ patients demonstrated significantly better periodontal indices than did those with fixed lingual appliances, which indicates a lower periodontal risk throughout treatment [10]. A comparison between Invisalign™ and vestibular appliances demonstrated conspicuous differences. Vestibular appliances caused greater more pain [11, 12], discomfort, greater analgesic consumption, and further functional and psychological disturbances during the initial stage of treatment [13, 14].

The body of literature assessing patient experiences during treatment is small, and most studies focus on pain and discomfort experiences either throughout- or post-treatment. Despite orthodontic literature having reported improved quality of life of orthodontic patients after orthodontic treatment [15–17], there is a decrease in QoL during treatment. These measurements have been made at different time points during treatment in various studies, creating confounding variables for analysis. It has been found that peak perceived discomfort with any orthodontic appliance occurs early in treatment [7, 18, 19]. Evaluation of patient experience during that period is important, as the health care industry focuses on enhancing patient experience [20].

To date, no studies have compared the QoL of patients being treated with vestibular, lingual, and aligner appliances. Many orthodontic surveys have employed questionnaires that are either non-validated or have a room for non-calibrated measurements. Currently, the most often used questionnaire in studies involving quality of life in health care assessment is WHOQOL-BREF, which is useful in epidemiological studies or clinical trials, as well as to evaluate the effectiveness of treatments.

The aim of this study was to evaluate the overall QoL using four domains (physical health, psychological well-being, social relationships, and environment) in adult orthodontic patients undergoing treatment in a private orthodontic practice. Compared were vestibular appliances, customized lingual orthodontic appliances, and clear aligners. The null hypothesis tested was no difference in QoL between the three appliance groups.

## Materials and methods

Approval from the Institutional Review Board (IRB) of the European University, DHCC, Dubai, UAE, was obtained for the study. The observational prospective cohort study was planned to determine QoL in adult orthodontic patients undergoing treatment with vestibular orthodontic appliances (022 Mini Clippy, TOMY, Japan), customized lingual appliances (Incognito, 3 M Unitek, Monrovia, USA), and clear aligner therapy (Flash Orthodontics Mumbai, India) during the first 6 to 9 weeks of orthodontic treatment.

### Sample

The samples were obtained prospectively from a single orthodontic office in Mumbai, Maharashtra, India, from January 2017 to March 2018. Forty-five consecutively treated adult patients within each appliance group were enrolled (i.e., 45 consecutively treated with vestibular appliances, 45 with lingual appliances, and 45 patients treated consecutively with aligners), resulting in a total sample of 135 patients. One hundred seventeen patients (86.7%) adequately answered the questionnaire; hence, our final sample was grouped into a vestibular group (*n* = 41), a lingual group (*n* = 37), and a clear aligner group (*n* = 39). The patients who participated in this study had the opportunity to choose either of the three appliances, after discussion with the treating orthodontist. The lingual and aligner treatments were approximately 50-60% more expensive than the conventional vestibular appliance.

Patients consented to participate in the study after having reviewed a written description with comprehensive information regarding the survey and an informed consent and information release form to be signed. Subjects had the opportunity to ask questions and clarify their concerns either verbally or by email if they desired any further information. Exclusion criteria were as follows: (1) severe crowding (> 8 mm) or extractions, (2) previous history of orthodontic treatment, (3) oral pathology, (4) significant medical history or medication usage, (5) use of auxiliaries within or prior to the study period (e.g., expanders, TPA, TADs), (6) patients younger than 18 years of age, or (7) if 20% or more of questionnaire data was missing from a survey form, i.e., not completed by the subject.

### Procedures

The WHOQOL–BREF questionnaire was used in the study. The questionnaire was administered at least 6 weeks (and up to a maximum of 9 weeks) after commencement of orthodontic appliance therapy, as patients experience the largest reduction in QoL during this timeframe [21]. This questionnaire was developed by the WHOQOL group with 15 international field centers, simultaneously, in an attempt to develop a quality of life assessment that would be applicable cross-culturally. The series of 26 validated questions divided into four domains were evaluated.

The steps in calibration of the data employed were as follows:

1. Checking all 26 items for an assessment from a range of 1-5.
2. Reversing three negatively phrased items (Q 3, Q4, and Q26).
3. Computation of domain scores based on scoring document guidelines.
4. Checking of domain scores and saving data sets for statistical analysis.

### Statistical analysis

Data analysis was carried out using the Statistical Package for Social Science (SPSS) computer software for Windows. A one-way ANOVA test was performed in order to compare the three groups for all four domains, i.e., physical health, psychological health, social relationships, and environment, and thereafter a Scheffe post hoc test was performed.

## Results

The three groups were observed for differences in age, gender, marital status, and educational level (Table 1). Homogeneity was demonstrated for gender, marital status, and educational level for all three groups. The age of the vestibular group (26.4) was significantly less than the lingual group (30), but was not significantly different to the aligner group (27.8).

**Table 1:** Pre-treatment variables

	Vestibular	Lingual	Aligner	V-L	V-A	L-A
				*P* signif.		
*n*	41	37	39			
Mean age (+−SD)	26.4 (+−7.3)	30 (+−6.9)	27.8 (+−6.9)	*P* < 0.05	NS	NS
Gender (male:female)	19:22	17:20	18:21	NS	NS	NS
Marital status (married:single)	14:27	11:26	13:26	NS	NS	NS
Education level (secondary:tertiary)	2:39	0:37	0:39	NS	NS	NS

Mean scores for domain 1, physical health, showed that the aligner group (28.1) had significantly greater scores than that of the vestibular (22.7) or lingual (22) groups. There was no statistical difference between the lingual and vestibular groups; however, the lingual group did score slightly less (Table 2).

**Table 2:** QoL of patients treated with vestibular, lingual, and aligner appliances

	Vestibular			Lingual			Aligner			V-L	V-A	L-A
	*n*	Mean	SD	*n*	Mean	SD	*n*	Mean	SD	*P* signif.		
Physical health (D1)	41	22.7	2.0	37	22	1.9	39	28.1	2.1	NS	< 0.001	< 0.001
Psychological health (D2)		15.2	1.9		18.4	2.2		23.2	2.0	< 0.001	< 0.001	< 0.001
Social relationships (D3)		7.8	1.4		10.2	1.2		10.9	1.5	< 0.001	< 0.001	NS
Environment (D4)		26.4	1.9		29.3	2.0		32.1	2.1	< 0.001	< 0.001	< 0.001
Overall		18	1.1		20.8	1.1		23.1	1	< 0.005	< 0.005	< 0.005

Domain 2, psychological health, demonstrated significant differences (*P* < 0.001) between all groups, with the aligner group scoring the highest (23.2), followed by the lingual (18.4) and vestibular (15.2) groups.

Domain 3, social relationship, showed that aligner (10.9) and lingual (10.2) scores were significantly greater (*P* < 0.001) than those of the vestibular group (7.8). There was no statistical difference between the aligner and lingual group, although the aligner group did score higher.

Domain 4, environment, displayed significant differences between all groups, with the aligner group scoring highest (32.1), followed by the lingual group (29.3), and lastly the vestibular group (26.4).

Overall, the highest mean score was obtained by the aligner group (23.1) and the lowest mean score was by the vestibular group (18). The mean domain scores for all three groups were significantly different (*P* ≤ 0.005) from each other (Table 2).

## Discussion

There were no statistically significant pretreatment variables (age, gender, marital status, and educational level) between any of the groups, with the exception of the age difference between the vestibular and lingual groups (26.4 vs 30). This does indicate that older patients tend to prefer more esthetic appliances, or that they can afford aligner or lingual treatment (which is more expensive at the clinic at which the study was performed). Cooper-Kazaz et al. [22] and Pacheco-Pereira et al. [23] observed a greater number of female patients choosing lingual or aligner therapy as their choice of appliance; however, we found no significant differences in gender in all three groups.

On evaluating specific domain scores, we observed that for domain 1 (which incorporates physical health attributes, i.e., daily activity, energy, fatigue, pain, medicine dependence, discomfort, sleep, rest, work capacity), patients in the lingual group scored 22 ± 1.9 and the aligner group scored 28.1 ± 2.1; the vestibular group had a score of 22.7 ± 2.0. This shows that aligner patients demonstrated improved physical health domain scores compared to both fixed appliance groups, and the results agree with Miller et al. [13], who concluded that vestibular appliances caused greater functional changes and pain, and White et al. [14] who found that vestibular appliances cause greater discomfort and require greater use of analgesics compared to aligners. Although there were no statistically significant differences between the lingual and vestibular groups in this domain, the vestibular group did have a higher score. These results confirm the results of Wu et al. [7, 8], who found greater oral impacts occurring within the lingual group, despite pain being similar albeit at different sites. Long et al. [6], in a systematic review comparing labial and lingual appliances, concluded that patients who underwent lingual orthodontic treatment were more likely to suffer from pain in the tongue and less likely to suffer from pain in the cheek and lip. Lingual appliances also increased the likelihood of speech difficulty.

Domain 2 refers to psychological attributes, i.e., body image, appearance, self-esteem, negative, or positive feelings. The aligner group outperformed the other two groups with a score of 23.2 ± 2.0. The lingual group (18.4 ± 2.0) was then followed by the vestibular group (15.2 ± 1.0). All these differences were significant. This result is consistent with the studies by Wu et al. [7, 8], who found a greater psychological well-being in lingual patients compared to vestibular patients. Aligner patients fared better than the other two groups, and this was also found by Miller et al. [13] who concluded that vestibular appliances caused greater decreases in psychosocial aspects compared to aligners. No other study has compared aligner patients during treatment to both vestibular and lingual patients for psychological impact; however, White et al.^14^ reported patients with traditional appliances having greater discomfort compared to aligner patients. Psychological adaption or a feeling of well-being/dysfunction plays an important role in the perception of orthodontic treatment by adult care seekers, and also subsequently recommending orthodontic therapy to potential care seekers. This aspect of orthodontic care and its quantification gains even more importance in the day and age of social media, where perceptions and experiences (both negative and positive) can be made readily available [24, 25]. It also helps professionals provide personalized treatment and improve standards of service and care, based on not just appliance efficacy and efficiency but also patient experiences. In fact, Noll et al. [26] found that Twitter users expressed more positive than negative sentiment about orthodontic treatment in general, with no significant difference in sentiment between vestibular appliances and aligners. This is at odds with our results which did find improved QoL for aligner patients. It should be noted that social media often does not reflect real life, and hence, what is posted may not be a true reflection of their experiences.

Domain 3 evaluated social relationships (both personal and social) and our sample showed comparable (*P* > 0.05) results between aligner (10.9 ± 1.5) and lingual (10.2 ± 1.2) groups, which scored significantly higher than the vestibular appliance group (7.8 ± 1.4). This, not surprisingly, reinforces the idea that social relationships are perceived to be better with esthetic appliances, as reported by previous studies [13].

Domain 4 evaluated environmental factors (financial, health and social care, home environment, etc.). The aligner group (32.1 ± 2.1) scored higher than the lingual group (29.3 ± 2.0) which in-turn scored higher than the vestibular group scored (26.4 ± 1.9). It is critical to consider while domain 4 is analyzed, that in an urban private practice setting in India, the patients choosing a lingual and an aligner appliance pay approximately 50-60% more than patients opting for treatment with vestibular appliances. This suggests a possible higher socio-economic status than patients in the vestibular group as cost of care could have an impact on the scores of domain 4.

The overall QoL scores for the three groups evaluated were in the following order: aligner (23.1 ± 1), lingual (20.8 ± 1.1), and vestibular (18 ± 1.1). This finding is consistent with White and colleagues [14] reporting that patient experience using aligners was superior. Pacheco-Pereira [23] also reported positive patient satisfaction in aligner patients. The majority of studies [14, 23, 27–29] have found that aligners have a lesser impact on QoL compared to other appliances, although a recent article by Antonio-Zancajo et al. [30] did find contradicting results. These results do not advocate the use of a particular appliance based solely on QoL. Efficiency and effectiveness of these appliances should be the primary factors taken into consideration when deciding on the most appropriate appliance.

Oral health is not solely linked to dysfunction or absence of oral disease; the concept includes the impact of oral conditions on self-confidence and social life. The effects of orthodontic treatment on quality of life have been previously measured and reviewed in a systematic review by Zhou et al. [15]. The included studies have shown that patients reported improvements in their QoL after orthodontic treatment [17, 31]. However, they also reported decreases in quality of life during treatment due to physical discomfort, including pain and functional limitations, and psychological discomfort. Psychological well-being has been shown to modulate a patient’s perceived improvement in QoL after orthodontic treatment [32].

## Limitations

The findings of the present study have to be carefully interpreted, especially when comparing them to existing literature. Factors such as quality of treatment, relationship with the orthodontist, and the impact of correction on QoL did not play a role in our assessments. Our aim was to simply assess the impact of the various appliances during orthodontic treatment on the QoL of patients. The present study had a response rate of 86.7%, which have introduced some confounding factors—in which way, remains uncertain. However, it should be taken into consideration that socio-cultural perceptions of quality of life could be different in other parts of the world. Future research could incorporate a uniform study design and questionnaires to a larger sample of practices. Multi-centric data perhaps would allow for global extrapolation.

## Conclusions

- ▪ Patients treated with aligners had significantly better QoL scores (28.1) for physical health than that of the vestibular (22.7) or lingual (22) appliances.
- ▪ The aligner group obtained significantly higher QoL (23.2) for psychological health, followed by the lingual (18.4) and vestibular (15.2) groups.
- ▪ Scores for social relationships indicated that aligner (10.9) and lingual (10.2) scores were significantly greater than those of the vestibular group (7.8).
- ▪ Domain 4 (environment) displayed significant differences between all groups, with the aligner group scoring highest (32.1), followed by the lingual group (29.3) and lastly the vestibular group (26.4).
- ▪ Overall, there were significant differences between all groups, with the highest QoL found in the aligner group (23.1), followed by the lingual group (20.8), and lastly, the vestibular group (18).

## Data Availability

The datasets used and/or analyzed during the current study are available from the corresponding author on reasonable request.

## Abbreviations

QoL: Quality of life
WHOQoL: The World Health Organization Quality of Life
